# Effects of red blood cells with reduced deformability on cerebral blood flow and vascular water transport: measurements in rats using time-resolved pulsed arterial spin labelling at 9.4 T

**DOI:** 10.1186/s41747-021-00243-z

**Published:** 2021-12-21

**Authors:** Adnan Bibic, Tea Sordia, Erik Henningsson, Linda Knutsson, Freddy Ståhlberg, Ronnie Wirestam

**Affiliations:** 1 Department of Medical Radiation Physics, Lund University, Lund, Sweden; 2 Centre for Mathematical Sciences, Lund University, Lund, Sweden; 3 Russell H. Morgan Department of Radiology and Radiological Science, Johns Hopkins University School of Medicine, Baltimore, MD USA

**Keywords:** Hemodynamics, Cerebrovascular circulation, Perfusion imaging, Magnetic resonance imaging, Erythrocytes

## Abstract

**Background:**

Our aim was to introduce damaged red blood cells (RBCs) as a tool for haemodynamic provocation in rats, hypothesised to cause decreased cerebral blood flow (CBF) and prolonged water capillary transfer time (CTT), and to investigate whether expected changes in CBF could be observed and if haemodynamic alterations were reflected by the CTT metric.

**Methods:**

Damaged RBCs exhibiting a mildly reduced deformability were injected to cause aggregation of RBCs. Arterial spin labelling (ASL) magnetic resonance imaging experiments were performed at 9.4 T. Six datasets (baseline plus five datasets after injection) were acquired for each animal in a study group and a control group (13 and 10 female adult Wistar rats, respectively). For each dataset, ASL images at ten different inversion times were acquired. The CTT model was adapted to the use of a measured arterial input function, implying the use of a realistic labelling profile. Repeated measures ANOVA was used (alpha error = 0.05).

**Results:**

After injection, significant differences between the study group and control group were observed for relative CBF in white matter (up to 20 percentage points) and putamen (up to 18–20 percentage points) and for relative CTT in putamen (up to 35–40 percentage points).

**Conclusions:**

Haemodynamic changes caused by injection of damaged RBCs were observed by ASL-based CBF and CTT measurements. Damaged RBCs can be used as a tool for test and validation of perfusion imaging modalities. CTT model fitting was challenging to stabilise at experimental signal-to-noise ratio levels, and the number of free parameters was minimised.

**Supplementary Information:**

The online version contains supplementary material available at 10.1186/s41747-021-00243-z.

## Key points

- Perfusion parameters were estimated by arterial spin labelling in a rat model.
- Red blood cells (RBCs) with reduced deformability were introduced for haemodynamic provocation.
- Effects of damaged RBCs were confirmed by differences in cerebral blood flow (CBF) between two groups.
- Haemodynamic effects of damaged RBCs were reflected by a prolonged capillary transfer time.
- The observation of significantly reduced regional CBF indicates that damaged RBCs may become a tool for validation of perfusion imaging techniques.

## Background

Arterial spin labelling (ASL) is a noninvasive magnetic resonance imaging (MRI) method, commonly used for measurement of brain perfusion, *i.e.*, cerebral blood flow (CBF), by employing magnetically labelled arterial water as an endogenous tracer [1, 2]. ASL CBF measurements are generally robust and have proven useful in the assessment of a number of diseases and conditions [3], for example cerebral infarction [4, 5], tumour evaluation [6, 7], epilepsy [8], and Alzheimer’s disease [9, 10]. Furthermore, as water is not a freely diffusible tracer in the brain [11–14], various methods for estimating the transcapillary exchange of cerebral blood water by ASL have been proposed [15–17].

The non-compartment model proposed by Kelly et al. [18] allows for the extraction of water transport time parameters, including capillary water transfer time (CTT), using data from an ordinary dynamic ASL experiment. The basic assumption is that labelled water is driven by a potential difference in large vessels, which causes the bulk flow, and when arriving at the microvascular space it distributes due to the random nature of the pseudo-diffusion motion in the capillary network. Finally, it exchanges between the capillary bed and the extravascular water through the blood-brain barrier. This transport is described by the general Langevin equation, which, in principle, enables separation of the local CTT (which includes both the pseudo-diffusion and filtration through the blood-brain barrier in the microvasculature) from the total transit time (which corresponds to the time it takes for water molecules to traverse the whole system) [18]. Successful application of the model is, however, challenging and further investigation and assessment of methodological robustness is indeed warranted.

The aims of this study were to use ASL CBF measurements, generally shown to be stable and robust [3], to corroborate effects of a proposed tool for haemodynamic challenge, as well as a to investigate whether the verified haemodynamic changes introduced to the system could also be captured by a modified version of a previously implemented transit-time modelling approach [19]. Hence, artificial modification of the deformability of red blood cells (RBCs), leading to increased blood viscosity and correspondingly increased microvascular resistance, was employed for controlled alterations of the haemodynamic conditions in the brain [20], hypothesised to reduce the CBF and increase the mean CTT. RBC deformability refers to the unique ability of the RBCs to change their shape in response to external stress, and normal RBC deformability is an important feature of the RBCs in order for them to be able to pass unimpeded through capillaries with a diameter smaller than the freely suspended RBC diameter. The increased viscosity caused by the reduction in deformability will impair the microcirculatory perfusion and oxygen delivery to peripheral tissue [21, 22], and RBCs with reduced deformability may also directly adhere to the endothelial membrane and block microvessels [23].

In this study, the effects on CBF and mean CTT [18, 19] in a group of adult rats subjected to injection of damaged RBCs, caused by glutaraldehyde exposure, were compared with the corresponding effects in a control group injected with normal RBCs. The experiments were designed to observe changes in haemodynamic conditions in each animal over time, before and after injection of RBCs. This study also reports further development of the non-compartment model for water transport [18, 19] by adopting a bolus-tracking ASL solution, including the use of a measured arterial input function instead of a theoretical rectangular input function.

## Methods

### General study design and experimental conditions

All experimental procedures were approved by the Local Committee for Animal Research Ethics (permit number M285-11) and performed according to the guidelines of the Swedish Animal Welfare Agency and in agreement with international guidelines. Female Wistar adult rats (Charles River Laboratories, Germany) with a weight of 220–250 g were used. Animals were housed on a 12-h light/dark cycle with *ad libitum* access to food and water. All experiments, including surgery and MRI, were conducted while the animals were anaesthetised. The sizes n_1_ and n_2_ of the control group (index 1) and the study group (index 2) were calculated using the formulas n_1_ = [(${\sigma}_1^2$ + ${\sigma}_2^2$/K)(1.96 + 0.84)^2^]/[|m_1_-m_2_|^2^] (for power = 0.8 and α = 0.05) and n_2_ = *K*·n_1_, where *K* is the enrolment ratio [24]. For the CBF endpoint the following input data were used: *K* =1.3, mean m_1_ = CBF_mean_~ 160 mL/100 g/min [25], expected mean m_2_ ~ 136 mL/100 g/min, ${\sigma}_1$ = ${\sigma}_2$ = SD_CBF_ ~ 20 mL/100 g/min [25]. Although sample size calculations are based on the primary endpoint, a supplementary calculation based on CTT was performed for corroboration, using *K* = 1.3, m_1_ = CTT_mean_ ~ 1.43 s [18], expected m_2_ = 1.57 s, ${\sigma}_1$ = ${\sigma}_2$ = SD_CTT_~ 0.12 s [18]. In accordance with the conditions of the ethical approval, all animals were euthanised immediately after the MRI experiments using a lethal dose of pentobarbital by intravenous or intraperitoneal injection.

### Animal anaesthetics, surgery and blood preparation

#### Animal anaesthetics

Anaesthetics were administered by inhalation through a locally manufactured mask, and anaesthesia was induced by 4% isoflurane and then maintained at 2.2% in a 1,000 mL/min oxygen mixture. Nitrous oxide (N_2_O), commonly used in an anaesthetic mixture, was avoided in this case because it is known to produce a significant increase in CBF in rats by up to 100% [26]. During the surgical procedure (normally taking between 20 and 30 min), an animal body temperature of 37 °C was maintained by an electrical heating pad.

#### Animal preparation

Animals were placed on the surgery table, and a 21G butterfly needle with catheter PE 50, filled up with heparin saline, was inserted into the lateral tail vein for transfusion of RBC suspension. Fresh blood was obtained from healthy anaesthetised donor rats (of the same provenience and characteristics as the rats in the CG/SG) by cardiac puncture. The blood from one donor rat (7–8 mL) was used for the preparation of the RBC suspensions for two transfusions.

#### Preparation of the red blood cells

The RBCs were separated from plasma by centrifugation at a relative centrifugal force of 1500G during 30 min. The RBCs were divided into two groups, *i.e.*, (*A*) suspension with normal RBCs, injected into the animals in the control group (CG) and (*B*) suspension with RBCs with reduced deformability, injected into the animals in study group (SG). To reduce the deformability of the cell membrane, the RBCs of group *B* were subjected to minimal hardening by incubation in 0.025% glutaraldehyde in phosphate-buffered saline at pH 7.4 during 30 min at room temperature. Following the incubation, the RBCs were washed in phosphate-buffered saline three times to completely remove the glutaraldehyde. The RBCs of group *A* were handled in the same way, but without adding the glutaraldehyde solution. The washed erythrocytes were diluted in the phosphate-buffered saline. Haematocrit was measured and adjusted by dilution to a level equal to that of the experimental rats [20].

### MRI experiments

For the MRI investigation, all animals were rapidly repositioned from the surgical table into an MRI animal holder, where the head of the rodent was secured using ear bars, bite bar and a nose cone to minimise motion during image acquisitions. The neck of each rat was also fixated with adhesive tape to reduce respiratory movement. The animal positioning, relative to the radiofrequency and gradient coils, was carefully considered to minimise animal-to-animal variability. Throughout the MRI experiments, breathing rate and oxygen saturation level were controlled, and the body core temperature was monitored by use of a rectal thermometer and maintained at 37 °C using warm air flowing into the magnet bore. The breathing rate was monitored with a MRI-compatible system (Model 1025, SAII, Stony Brook, NY, USA) using a respiratory air-filled pressure sensor placed in contact with the abdomen, and the breathing rate was controlled by adjusting the depth of anaesthesia to maintain a stable respiration rate around 60 min^−1^.

**Fig. 1:**
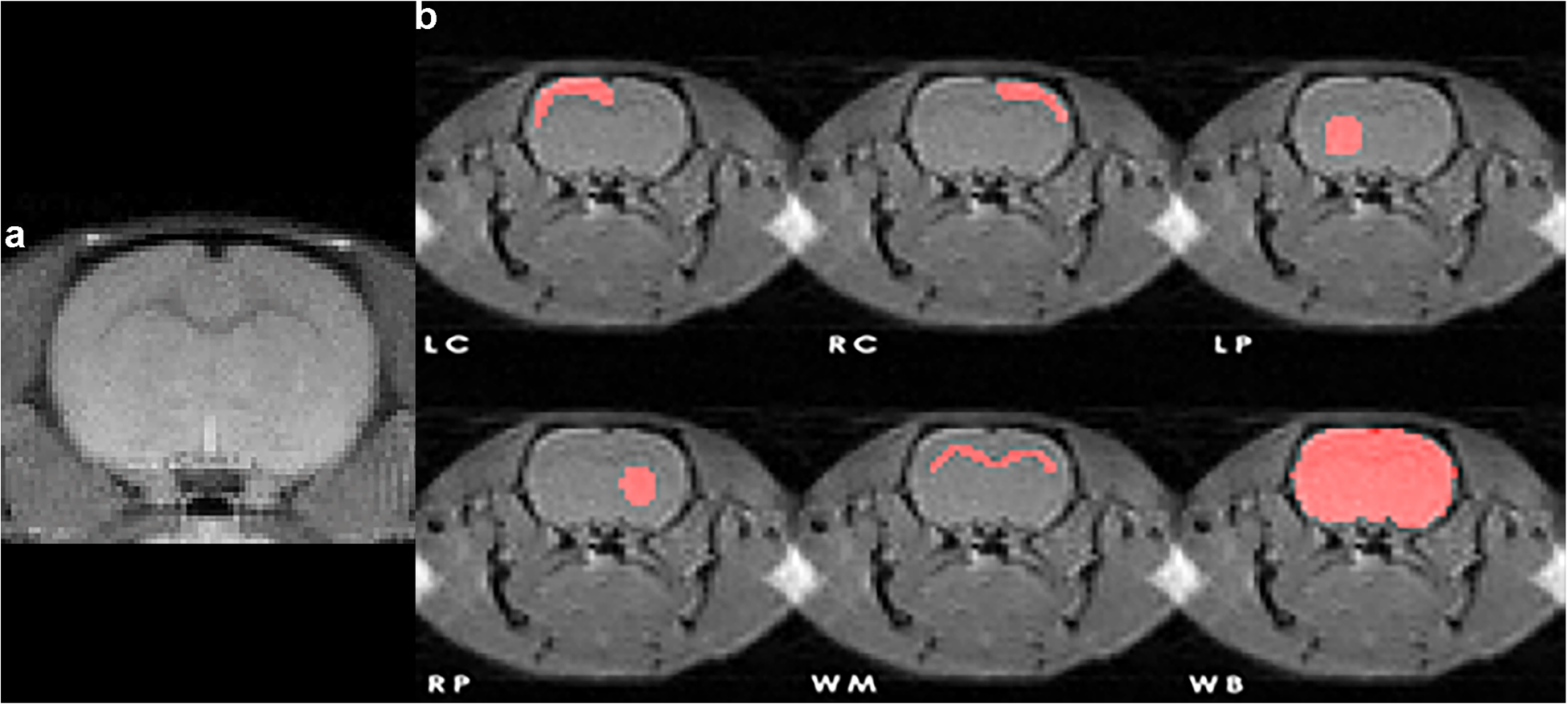
**a** Fast spin echo anatomical image showing the location of the slice under investigation. **b** Regions of interest (ROIs) used for quantification of average cerebral blood flow (CBF) and capillary transfer time (CTT). The ROIs are overlaid on the slice-selective ASL echo-planar images. *LC* Left cortex, *RC* Right cortex, *LP* Left putamen, *RP* Right putamen, *WM* White matter, *WB* All brain tissue in the selected slice

The MRI experiments were performed using a 9.4 T horizontal bore animal scanner (Agilent Inc., Palo Alto, CA, USA) with the 205/120 HD (High Duty Cycle) gradient coil. A 72-mm inner diameter volume coil was used for radiofrequency transmission, and the signal was received using a 4-channel array head coil. T_2_-weighted fast spin echo anatomical images were used for slice localisation. To standardise the scan procedures, the imaging slice of interest was positioned just frontal to the bregma, which is an easily recognisable structure in MRI images, located at the most forward crossing fibre of the anterior commissure [27] (Fig. 1a). A single-slice flow-sensitive alternating inversion recovery ASL sequence with a three-shot segmented spin-echo echo-planar imaging readout was implemented with the following parameters: field of view 32 mm^2^; matrix 64^2^; slice thickness 1 mm; repetition time 5.1 s; echo time 10 ms; spectral width 250 kHz; echo spacing 0.32 ms; tag region width 5 mm (to ensure that there was no mismatch between the effect of the slice selective and the global inversion pulses on the imaging slice [28]). A hyperbolic secant adiabatic inversion pulse with a bandwidth of 20 kHz was used for both the slice selective and global inversions. To minimise the signal from the static tissue, two pre-saturation pulses were applied in the slice of interest.

For each dataset, ASL images at ten different time points (corresponding to different inversion times) were acquired for model fitting according to Eq. 2, starting at inversion time (TI) 300 ms followed by a 300 ms increment between time points. Before the injection of RBCs, one ASL dataset was acquired, in both CG and SG rats, to be used as baseline, and after injection totally five ASL datasets were acquired for each animal in both groups. Hence, in the analysis (described below), six datasets for each animal were evaluated separately, *i.e.*, an initial pre-injection baseline measurement (assigned time *t* = 0) and five postinjection measurements. Considering that the biological half-life of the damaged RBCs was assumed to be approximately 13 min [20], the first postinjection data point was acquired 10 min after injection and subsequent postinjection data points were acquired in 11 min and 30 s intervals.

Tissue T_1_, as required for the modelling according to Eq. 2, was estimated separately on a region of interest (ROI) basis in a location corresponding to the area used in the ASL measurements, using the inversion recovery fast spin-echo imaging method. The parameters used for T_1_ estimation were as follows: repetition time 5.1 s; TI 0.022, 1, 2, 3, 4, 5 s; effective echo time 28 ms; echo-spacing time 14 ms; echo train length 4; number of excitations 4. The first echo was assigned to the centre of *k*-space.

### Theory and postprocessing procedure

Labelled and control images were pair-wisely subtracted to attain perfusion-weighted ASL-MRI images, and these difference maps were denoised by wavelet-domain filtering [29] using an in-house software program written in IDL 7.1 (ITT Visual Information Solutions, Boulder, CO, USA).

The CBF values were calculated using a general kinetic model [2] according to the Buxton approach (Eq. 1):

1 $$ \Delta  M(t)=\left\{\begin{array}{cc}0& 0<t\le BAT\\ {}\frac{2{M}_{0B}\bullet CBF\bullet \alpha }{\Delta R}{e}^{-\frac{(BAT)}{T_{1b}}}\bullet \left(1-{e}^{-\Delta R\left(t- BAT\right)}\right)\bullet {e}^{\frac{-\left(t- BAT\right)}{T_{1 app}}}& BAT<t\le BAT+ BL\\ {}\frac{2{M}_{0B}\bullet CBF\bullet \alpha }{\Delta R}{e}^{-\frac{(BAT)}{T_{1b}}}\bullet \left(1-{e}^{-\Delta R\bullet BL}\right)\bullet {e}^{\frac{-\left(t- BAT\right)}{T_{1 app}}}& BAT+ BL<t\end{array}\right. $$

where ∆*M* is the magnetisation difference (obtained from difference maps), *T*_1*b*_ [= 2.2 s at 7 T [30]] is the spin-lattice (longitudinal) relaxation time of blood, *M*_0*B*_ is the equilibrium magnetisation of arterial blood, *α* is the inversion efficiency (assumed to be 1), BAT is the bolus arrival time, BL is the bolus length, Δ*R* is *R*_1*b*_*- R*_1app_, where *R*_1b_ is the relaxation rate of blood (*R*_1*b*_=1/*T*_1*b*_ = 1/2.2 s), and *R*_1app_= 1/*T*_1app_ is the apparent relaxation rate measured under the inflow of fresh blood magnetisation. Values of *M*_0B_, BAT, BL, and *T*_1app_ were all estimated by fitting the Buxton model to the dynamic, multi-TI, experimental ASL-MRI data.

The relative arterial transit time (rATT) and the mean capillary transfer time (CTT) were extracted by fitting the model in Eq. 2, which was adapted to allow for the use of an experimental arterial input function, according to Appendix.

2 $$ \Delta  M(t)=\frac{rTTT\bullet S}{\sqrt{4\pi \bullet CTT}}{\int}_0^t\Delta  {M}_a\left(\tau \right)\frac{e^{-\frac{t-\tau }{T1}}}{{\left(t-\tau \right)}^{3/2}}\mathit{\exp}{\left(-\frac{rTTT-\left(t-\tau \right)}{\sqrt{4\bullet CTT\bullet \left(t-\tau \right)}}\right)}^2 d\tau $$

where rTTT is the relative total transit time (rTTT = rATT + CTT), Δ*M*_*a*_ is the measured arterial concentration (*i.e.*, the arterial input function), registered in a large artery in the same slice as the tissue concentrations curves. *S* is a scaling factor which accounts for proportional errors in the arterial concentration estimation, owing to issues such as spatial variations in coil sensitivity, differences in equilibrium magnetisation, differences in T_2_ and arterial partial volume effects. To obtain a reasonable estimate of *S*, preliminary fittings were conducted with S as a free parameter. In the final analysis, to prevent overfitting, *S* was taken as the mean value over all pre- and post-injection time points for each animal, obtained from the preliminary fitting procedure. T_1_ is the longitudinal relaxation time of the tissue, calculated by least-squares fitting of Eq. 3 to the experimental inversion recovery MRI signal from each ROI:

3 $$ {M}_Z(TI)={M}_0\bullet \left(1-2\alpha \bullet {e}^{\frac{- TI}{T_1}}+{e}^{\frac{- TR}{T_1}}\right) $$

where *M*_*Z*_ (TI) is the magnitude MR signal collected at each TI, *α* is the inversion efficiency and *M*_0_ is the fully relaxed magnetisation. *M*_0_, α and T_1_ are fitted parameters.

The underlying fitting process was performed in MATLAB (R2018a, The MathWorks, Inc., Natick, MA, USA) using FMINUIT (version 2.3.1, Giuseppe Allodi, Dipartimento di Fisica, Universita di Parma, Italy), which is based on MINUIT (CERN, Geneva, Switzerland) [31].

### ROI-based analyses

Regional CBF and CTT values were calculated, on a ROI basis, for six predefined cerebral ROIs, corresponding to the left cortex, right cortex, left putamen, right putamen, and white matter. A ROI including the total brain tissue in the chosen slice was assumed to represent global brain levels and is referred to as the whole brain (WB) estimate below (see Fig. 1). ROIs were placed by an experienced preclinical MRI scientist (A.B.). Results were reported as absolute baseline levels in combination with relative changes after injection of damaged RBCs, as this facilitates a direct comparison of the effects (in terms of magnitude and variability) on CBF with the corresponding effects on CTT.

### Statistical analysis

Statistical analysis was accomplished using a repeated measures ANOVA test (*α* = 0.05) to test for effect of group (CG *versus* SG) and effect of time after injection (XLSTAT, Addinsoft, Paris, France). A limited number of planned observations were conducted, and no correction for multiple comparisons was required [32]; *p* values are reported, which enables readers to apply an alternative approach for estimating a different level of inference.

## Results

### Study population and monitoring of vital parameters

Based on the sample size calculations, 23 rats were included in the MRI study: 13 in the study group (SG) and 10 in the control group (CG). No abnormal readings of vital parameters (specified above) were observed during the course of the MRI experiments. The oxygen saturation was always above 95%, and no adjustment of oxygen concentration was needed.

### T_1_ measurements

The mean T_1_ estimates in the investigated ROIs were 1.61 s (WM), 1.75 s (putamen), 1.78 s (cortex) and 1.75 s (WB), with standard deviations (SDs) ranging between 0.03 s and 0.08 s (Table 1). The obtained mean T_1_ value for the respective region was used, when applying Eq. 2, for all measurements.

**Table 1:** T1 estimates in different brain regions

Region of interest	Mean T1 (s)	Standard deviation (s)
Left cortex	1.78	0.08
Right cortex	1.78	0.09
Left putamen	1.75	0.07
Right putamen	1.75	0.07
White matter	1.61	0.03
Whole brain	1.75	0.08

### Changes in cerebral blood flow

**Fig. 2:**
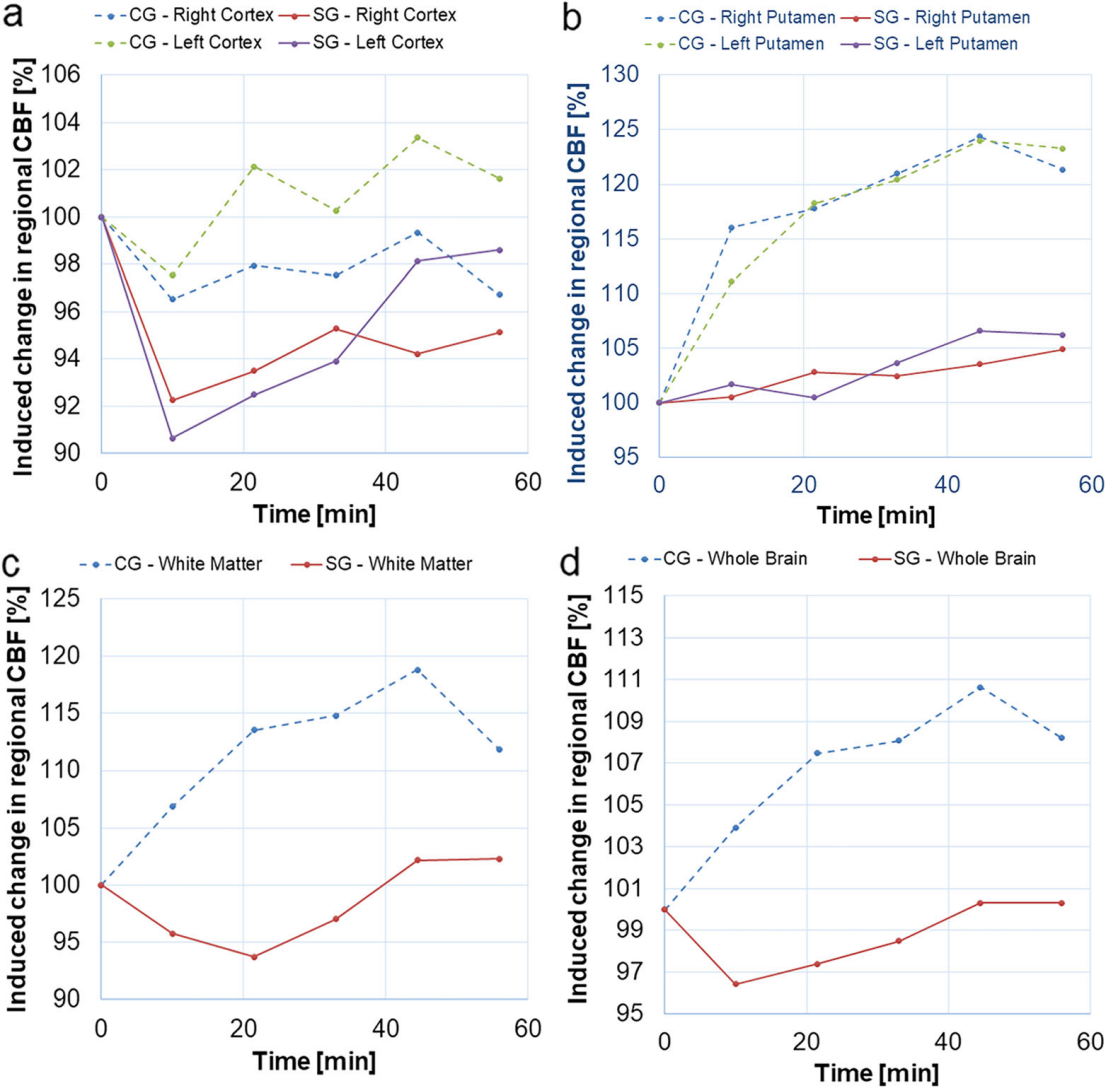
Relative cerebral blood flow (CBF) in (**a**) the left and right cortex, (**b**) the left and right putamen, (**c**) white matter, and (**d**) whole brain, prior to and after injection of damaged (study group, SG) and normal (control group, CG) red blood cells

**Fig. 3:**
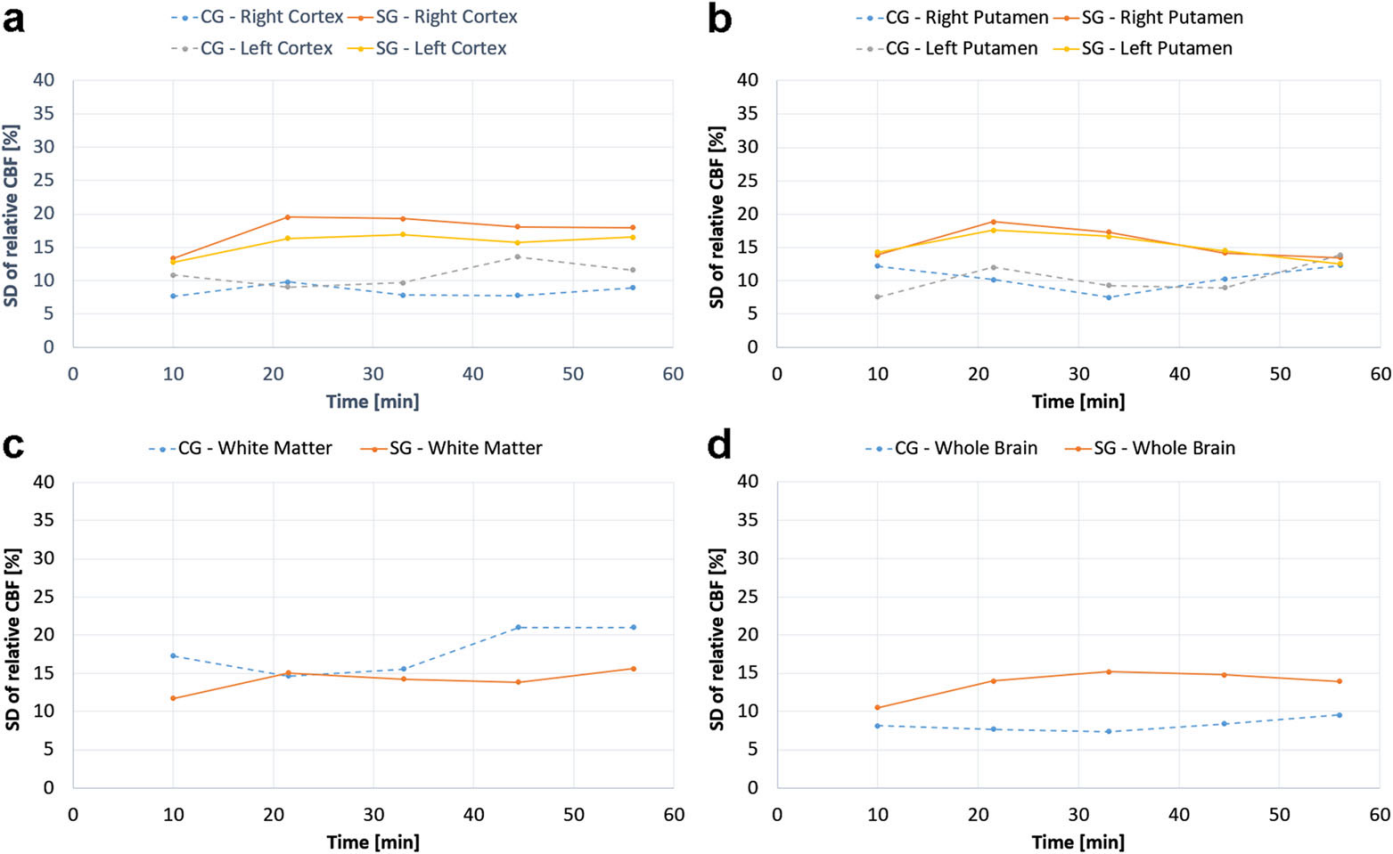
Standard deviation of relative cerebral blood flow (CBF) estimates in (**a**) the left and right cortex, (**b**) the left and right putamen, (**c**) white matter, and (**d**) whole brain, prior to and after injection of damaged (SG) and normal (CG) red blood cells

The normalised mean values of one global (WB) and five regional CBF estimates are illustrated in Fig. 2. For left and right cortex (Fig. 2a), the injection of damaged RBCs resulted in a decrease in relative CBF to around 90− 92% of preinjection levels, followed by a slow recovery towards a normal level. No significant differences in relative regional CBF were found between the CG and the SG. In the left and the right putamen (Fig. 2b, Table 2), relative CBF values were significantly different between the two groups. The putamen regional CBF increased in both groups, but significantly more in the control group. With regard to WM (Fig. 2c, Table 2), the relative CBF showed a significant difference between the two groups. The regional CBF in WM in the CG increased to 119% of the preinjection level at the fourth post injection time point, and it decreased slightly to 94% in the SG at the second postinjection time point. The global CBF changes (WB) tended to differ markedly between groups at the second and fourth postinjection time points (Fig. 2d). The global CBF decreased slightly to 96% of the preinjection level immediately after injection of the damaged RBCs and then returned to the normal level at the fourth post-injection time point. In the CG, CBF increased, with a maximum at the fourth postinjection time point, to 119% of the preinjection level. As an overall observation, the largest differences between the two groups occurred approximately 20 min after the injection of RBCs. A slight systematic tendency was that the CBF difference between groups increased soon (10–20 min) after the injection and then slightly decreased towards the end of the measurement series. The mean baseline CBF values, and the corresponding SDs, for different brain regions are summarised in Table 3. The SDs of relative CBF values in CG and SG are displayed in Fig. 3. All absolute mean CBF values and the corresponding SDs are provided in Additional file 1: Table S1.

**Table 2:** Results of repeated measures ANOVA test (*p*-values)

Region of interest	Dependent variable			
	Cerebral blood flow		Capillary transfer time	
	Test for between subject effects (effect of group)	Test for within subject effects (effect of time after injection)	Test for between subject effects (effect of group)	Test for within subject effects (effect of time after injection)
Left cortex	0.266	0.009	0.282	0.605
Right cortex	0.543	0.724	0.816	0.863
Left putamen	0.006	< 0.001	0.007	0.045
Right putamen	0.004	0.025	0.037	0.100
White matter	0.020	0.005	0.316	0.071
Whole brain	0.065	0.001	0.135	0.338

**Table 3:** Baseline cerebral blood flow and capillary transfer time estimates (mean ± standard deviation, *n* = 23) in different brain regions

Region of interest	Cerebral blood flow (mL/100 g/min)	Capillary transfer time (s)
Left cortex	220 ± 75	0.98 ± 0.36
Right cortex	213 ± 72	1.16 ± 0.39
Left putamen	193 ± 60	1.05 ± 0.39
Right putamen	190 ± 61	1.17 ± 0.50
White matter	139 ± 53	1.27 ± 0.57
Whole brain	197 ± 59	0.94 ± 0.30

### Changes in capillary transfer time

**Fig. 4:**
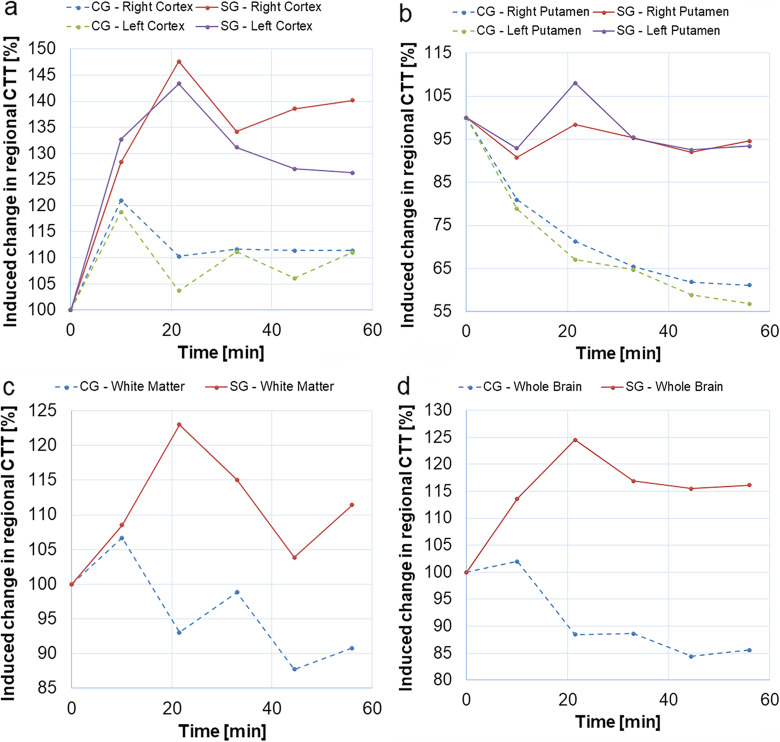
Relative capillary transfer time (CTT) in (**a**) the left and right cortex, (**b**) the left and right putamen, (**c**) white matter, and (**d**) whole brain, prior to and after injection of damaged (study group, SG) and normal (control group, CG) red blood cells. Data were obtained using the model in Eq. 2

**Fig. 5:**
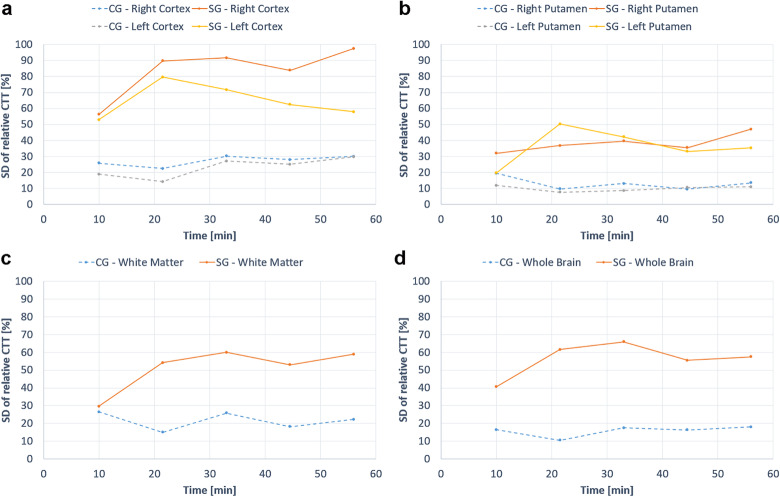
Standard deviation of relative capillary transfer time (CTT) estimates in (**a**) the left and right cortex, (**b**) the left and right putamen, (**c**) white matter, and (**d**) whole brain, prior to and after injection of damaged (SG) and normal (CG) red blood cells

The normalised CTT estimates (obtained using the model in Eq. 2) in cortex, putamen and white matter, as well as for all brain tissue in the selected slice (WB), are shown in Fig. 4. Generally, differences in relative CTT between CG and SG were visually observed in all ROIs (with longer CTT in the SG in all cases). The SG CTT in left and right cortex (Fig. 4a), WM (Fig. 4c) and in the WB ROI (Fig. 4d) tended to increase after injection of damaged RBCs, but the differences between groups did not reach statistical significance. In left and right putamen (Fig. 4b, Table 2), the differences between groups were statistically significant, and CTT remained unchanged in the SG after injection but decreased in the CG. The mean baseline CTT values, and the corresponding SDs, for different brain regions, are summarised in Table 3. The SDs of relative CTT values in CG and SG are provided in Fig. 5. All absolute mean CTT values and the corresponding SDs are provided in Additional file 1: Table S1.

## Discussion

In the present study, RBCs with reduced deformability were shown to have a significant effect on CBF over time after injection, in parts of the brain, compared with a control group. By using a robust ASL metric (*i.e.*, CBF), we thus confirmed that damaged RBCs had a measurable effect on the haemodynamics. This, in turn, allowed for an attempt to investigate whether our improved non-compartment model, accommodating a measured arterial input function (Eq. 2), showed the ability to capture corresponding changes in CTT (related to the combination of microvascular passage and transcapillary permeability). Significant CTT changes over time after injection were observed in the putamen, which is indeed promising, but the variability was generally large. A trend towards a decrease in CBF (relative to the normalised baseline CBF) was observed in the SG initially after injection of RBCs with reduced deformability (Fig. 2, see, for example, WB). Relative CBF after RBC injection was generally lower in the study group, and the most pronounced differences between groups, after injection, were seen in the putamen and in the WM (15–20 percentage points difference between groups). This observation is in agreement with previously measured blood velocity in an artificial microvascular network when RBCs were damaged using 0.025% glutaraldehyde concentration [33]. The CBF difference between groups was moderately lower in the cortex (5–10 percentage points). Generally, an increase in CBF was observed in the control group, after the injection, and this effect is most likely explained by the fact that the increased total blood volume (caused by the injection) induced a higher blood pressure which may cause increased CBF [34]. Depending on the mode of blood viscosity alteration and the degree of accompanying change in oxygen content, changes in CBF can either be clearly observable [35–37], or to a large extent counteracted by the cerebral autoregulation mechanism [38, 39]. Damaged RBCs as a means of changing the viscosity has previously been shown to result in ~ 10% reduction of CBF in rats [20], but other mechanisms than the trapping of damaged RBCs may also play a role, for example, increased platelet adhesion to the vessel walls [40].

With regard to relative CTT, a fixed scaling factor in the model (Eq. 2) was employed as a regularisation to increase stability in the fitting algorithm. The fixed scaling factor *S* was approximated by the mean value over all preinjection and postinjection time points, obtained from a preceding preliminary fitting procedure, and it was calculated separately for all ROIs in each animal. The use of the mean scaling factor as a fixed value may introduce a bias in the CTT (most commonly a slight overestimation), but when using the estimates to visualise relative changes in CTT over time the approach is still likely to improve the precision. It might, however, be reasonable to interpret the estimates as ‘apparent’ CTTs at this point. ASL suffers from inherently low signal-to-noise ratio, necessitating a limited spatial resolution (large voxels) with an associated risk of partial volume effects (*e.g.*, in cortex and WM) while still prohibiting a robust pixel-by-pixel analysis of CTT. This is reflected by large SDs in relative CTT even at the ROI level, particularly in the study group (see Fig. 5). Analysis of CTT based on the mean signal within a ROI may also lead to a bias caused by averaging of data from intra-ROI voxels which may show a spatially varying arrival time (*i.e.*, varying ATTs). Hence, if a ROI includes voxels with different ATTs, the model may capture the effects of this ATT distribution as a diffusion component, and this would, in such a case, be translated into an overestimation of CTT. This, again, emphasises that caution should be exercised in the numerical interpretation of the current CTT estimates. The injection of deformed RBCs induced a 25% relative increase in CTT for the whole slice. Significant differences between SG and CG were observed in the left and right putamen (see Table 2), and a longer relative CTT was observed in the SG compared with the CG, as hypothesised. There was a visually observable difference in relative CTT estimates between the two groups also in other ROIs, but the differences did not reach statistical significance. Interestingly, the CTTs were considerably shortened in putamen and white matter in the CG, correlating with increased CBF in the same ROIs. These effects might be caused by the injection of a large additional systemic blood volume, as mentioned above. The effects of an elevated arterial blood pressure are counteracted by cerebrovascular constriction (vasoconstriction) to maintain the CBF by autoregulation. When autoregulation reaches its upper limit, the microvascular blood volume is still decreased while CBF starts to increase (as observed in this study), and this situation is potentially associated with damage to the blood-brain barrier. Increased CBF and decreased microvascular blood volume entails shorter transit times, according to the central volume theorem (and shorter CTT would also be expected in the potential case of a damaged blood-brain barrier according to the theory behind the model in Eq. 2).

The mean baseline CBF levels (Table 3) were in reasonable to good agreement with previous ASL measurements in rats, *i.e.*, 170–177 mL/100 g/min [25] to 240–250 mL/100 g/min [41] in motor/sensorimotor/sensory cortex, 250 mL/100 g/min in thalamus [41], 157 mL/100 g/min in caudate putamen [25], 148 mL/100 g/min in WM [42], and 158 mL/100 g/min across a whole slice [43]. The mean baseline CTT values obtained in this study (Table 3) were in line with, or slightly below, values previously published by Kelly et al. [18], who reported CTTs ranging from 1.27 s to 1.66 s. Reported CTT values in humans have generally been substantially shorter [19].

Previously proposed methods for controlled modification of CBF include, for example, CO_2_-related dilation or constriction of cerebral arteries and arterioles entailing an reversible effect on CBF, where hypercapnia leads to increased CBF while hypocapnia decreases the CBF [44, 45], global ischemia using a blood-vessel-occlusion model for induced hypoperfusion [46] and the use of pharmacological manipulation, such as acetazolamide [46] (which alters brain CBF without altering brain metabolism) or methylphenidate [47]. However, blood-vessel-occlusion methods are highly invasive and require substantial efforts from well-trained personnel, and pharmacological manipulations have generally a small effect on CBF. Advantages of using RBCs with reduced deformability may be found, for example, in reproducibility tests of haemodynamic measurements or as a stroke model where the decreased RBC deformability results in microvascular occlusion [20], which in turn may result in tissue necrosis [48, 49]. Hence, supplemental methods to change the cerebral haemodynamics under controlled conditions are generally warranted, in order to test or validate perfusion measurement methods, for example, in medical imaging. In this study, it seems reasonable to view the results of well-established and stable ASL CBF measurements [50] as an indication that RBCs with artificially reduced deformability do in fact alter the microvascular properties in a preclinical setting. This initial establishment of effects on the haemodynamics, caused by the RBCs, would then, in the next step, imply that the more recently introduced CTT parameter does, indeed, show some initial promise in its ability to register haemodynamic alterations affecting the microcirculation.

With regard to limitations, one source of uncertainty in this study is the use of anaesthesia, which is necessary to avoid movement artefacts during image acquisition but also, obviously, to prevent the animals from suffering after the surgical procedure. Isoflurane is a cerebral vasodilator, known to affect basal cerebral haemodynamics, and it has been shown that the CBF in anaesthetised rats is markedly higher than in the awake state (20% to 70% across different brain conditions) [51]. Body temperature, blood pressure, respiratory rate, PaO_2_, PaCO_2_ and pH are other factors that can alter CBF [52], and these factors might, in principle, have been affected by the procedures performed on the rats. It should, however, be noted that no differences between groups with regard to respiration rate or other monitored vital parameters were observed in this study. Furthermore, the lack of histological examination may pose a potential limitation to the comparison of obtained data with reference standards.

As a final clinical and translational outlook, one might mention that haemorheological abnormalities, particularly RBC deformability changes, have been shown to correlate with severe complications of several haematological diseases such as malaria, hereditary spherocytosis, diabetes mellitus and paroxysmal nocturnal haemoglobinuria [53–57]. Due to RBC dehydration, sickle cell disease is specifically associated with a decrease in erythrocyte deformability; due to impaired ability to flow through the microcirculation, sickle red blood cells tend thus to cause vascular occlusive episodes, ischemia and infarction [49, 58]. Hence, *in vivo* techniques to monitor effects of impaired RBC deformability would be of interest, and ASL-based observations of the hemodynamic impact of mildly damaged RBCs are thus of potential relevance also from a clinical perspective.

In conclusion, haemodynamic changes caused by injection of RBCs with mildly reduced deformability were observed by ASL-based CBF and CTT measurements. The well-established CBF measurements indicate that damaged RBCs can be used as a tool for test and validation of perfusion imaging modalities and haemodynamic parameters. The CTT model (Eq. 2) is likely to suffer from a lack of precision at experimental signal-to-noise ratio levels, and stabilisation of the CTT model fitting required special attention, but promising results with respect to registered CTT changes after the established haemodynamic challenge, caused by the damaged RBCs, were still obtained.

### Supplementary Information

**Additional file 1: Table S1.** Absolute mean CBF and CTT values, and the corresponding standard deviations, for all investigated regions of interest and all points in time, for control group (CG) and study group (SG).

## Data Availability

The datasets used and analysed during the current study are available from the corresponding author on reasonable request.

## Appendix

Consider the Fokker-Planck equation (A1)

A1 $$ \frac{\partial c}{\partial t}=-F\frac{\partial c}{\partial V}+P\frac{\partial^2c}{\partial {V}^2}-\frac{c}{T_1},V\in R,t\ge 0 $$

and its bolus-tracking solution (A2) (i.e., Eq. 19 in Ref. [18]):

A2 $$ c\left(V,t\right)={\int}_0^t{c}_0\left(\tau \right)\frac{\exp \left(-\frac{t-\tau }{T_1}\right)}{\sqrt{4\pi\ P\ \left(t-\tau \right)}}\frac{V}{\left(t-\tau \right)}\exp \left(-\frac{{\left(V-F\left(t-\tau \right)\right)}^2}{4\ P\left(t-\tau \right)}\right) d\tau $$

where F is blood flow and P represents the diffusion component (i.e., pseudo-diffusion and the filtration through the blood-brain barrier). With the following boundary conditions, *c*(0, *t*) = *c*_*a*_(*t*) = 0 for *t* < 0 and *t* > *t*_*b*_, the solution to (A2) becomes

A3 $$ c\left(V,t\right)=\frac{V}{\sqrt{4\pi\ P\ }}{\int}_0^{\min \left(t,{t}_b\right)}{c}_a\left(\tau \right)\frac{\exp \left(-\frac{t-\tau }{T_1}\right)}{{\left(t-\tau \right)}^{\frac{3}{2}}}\mathit{\exp}\left(-\frac{{\left(V-F\left(t-\tau \right)\right)}^2}{4\ P\left(t-\tau \right)}\right) d\tau $$

By defining the relative total transit time rTTT $=\frac{V}{F}$ , $CTT=\frac{P}{F^2}$ , $\frac{F^2}{P}=\frac{1}{CTT}$ and $\frac{V^2}{P}=\frac{F^2\bullet {rTTT}^2}{F^2\bullet CTT}=\frac{rTTT^2}{CTT}$, then the general solution will be given as

A4 $$ c\left(t, rTTT, CTT\right)=\frac{rTTT\bullet S}{\sqrt{4\ \pi\ CTT}}{\int}_0^{\min \left(t,{t}_b\right)}{c}_a\left(\tau \right)\frac{\exp \left(-\frac{t-\tau }{T_1}\right)}{{\left(t-\tau \right)}^{\frac{3}{2}}}\mathit{\exp}\left(-\frac{{\left( rTTT-\left(t-\tau \right)\right)}^2}{4\  CTT\left(t-\tau \right)}\right) d\tau $$

where *S* is a scaling factor aiming to correct for proportional errors in the registration of arterial concentration, such as partial volume effects, M_0_ differences and different T_2_ in blood and tissue.

## Abbreviations

ASL: Arterial spin labelling
CBF: Cerebral blood flow
CG: Control group
CTT: Capillary transfer time
MRI: Magnetic resonance imaging
rATT: Relative arterial transit time
RBC: Red blood cell
ROI: Region of interest
rTTT: Relative total transit time
SD: Standard deviation
SG: Study group
TI: Inversion time
WB: Whole brain
WM: White matter

## References

[1] Detre JA, Leigh JS, Williams DS, Koretsky AP (1992). Perfusion imaging. Magn Reson Med.

[2] Buxton RB, Frank LR, Wong EC, Siewert B, Warach S, Edelman RR (1998) A general kinetic model for quantitative perfusion imaging with arterial spin labeling. Magn Reson Med 40:383–396. 10.1002/mrm.1910400308 10.1002/mrm.1910400308 9727941

[3] Wintermark M, Sesay M, Barbier E, Borbély K, Dillon WP, Eastwood JD, Glenn TC, Grandin ĆB, Pedraza S, Soustiel JF, Nariai T, Zaharchuk G, Caillé JM, Dousset V, Yonas H (2005). Comparative overview of brain perfusion imaging techniques. Stroke.

[4] Wolf RL, Alsop DC, McGarvey ML, Maldjian JA, Wang JJ, Detre JA (2003). Susceptibility contrast and arterial spin labeled perfusion MRI in cerebrovascular disease. J Neuroimaging.

[5] Detre JA, Alsop DC, Vives LR, Maccotta L, Teener JW, Raps EC (1998). Noninvasive MRI evaluation of cerebral blood flow in cerebrovascular disease. Neurology.

[6] Noguchi T, Yoshiura T, Hiwatashi A, Togao O, Yamashita K, Nagao E, Shono T, Mizoguchi M, Nagata S, Sasaki T, Suzuki SO, Iwaki T, Kobayashi K, Mihara F, Honda H (2008). Perfusion imaging of brain tumors using arterial spin-labeling: correlation with histopathologic vascular density. AJNR Am J Neuroradiol.

[7] Warmuth C, Gunther M, Zimmer C (2003). Quantification of blood flow in brain tumors: Comparison of arterial spin labeling and dynamic susceptibility-weighted contrast-enhanced MR imaging. Radiology.

[8] Pendse N, Wissmeyer M, Altrichter S, Vargas M, Delavelle J, Viallon M, Federspiel A, Seeck M, Schaller K, Lövblad KO (2010). Interictal arterial spin-labeling MRI perfusion in intractable epilepsy. J Neuroradiol.

[9] Alsop DC, Detre JA, Grossman M (2000) Assessment of cerebral blood flow in Alzheimer’s disease by spin-labeled magnetic resonance imaging. Ann Neurol 47(1):93–100. PMID: 10632106 10632106

[10] Wolk DA, Detre JA (2012). Arterial spin labeling MRI: an emerging biomarker for Alzheimer’s disease and other neurodegenerative conditions. Curr Opin Neurol.

[11] Eichling JO, Raichle ME, Grubb RL, Ter-Pogossian MM (1974). Evidence of the limitations of water as a freely diffusible tracer in brain of the rhesus monkey. Circ Res.

[12] Ginsberg MD, Busto R, Harik SI (1985). Regional blood-brain barrier permeability to water and cerebral blood flow during status epilepticus: insensitivity to norepinephrine depletion. Brain Res.

[13] Silva AC, Williams DS, Koretsky AP (1997). Evidence for the exchange of arterial spin-labeled water with tissue water in rat brain from diffusion-sensitized measurements of perfusion. Magn Reson Med.

[14] Herscovitch P, Raichle ME, Kilbourn MR, Welch MJ (1987). Positron emission tomographic measurement of cerebral blood-flow and permeability surface-area product of water Using [15O] water and [11C] butanol. J Cereb Blood Flow Metab.

[15] Parkes LM, Tofts PS (2002). Improved accuracy of human cerebral blood perfusion measurements using arterial spin labeling: accounting for capillary water permeability. Magn Reson Med.

[16] Li KL, Zhu X, Hylton N, Jahng GH, Weiner MW, Schuff N (2005). Four-phase single-capillary stepwise model for kinetics in arterial spin labeling MRI. Magn Reson Med.

[17] Petitclerc L, Schmid S, Hirschler L, van Osch MJP (2021). Combining T2 measurements and crusher gradients into a single ASL sequence for comparison of the measurement of water transport across the blood-brain barrier. Magn Reson Med.

[18] Kelly ME, Blau CW, Kerskens CM (2009). Bolus-tracking arterial spin labelling: theoretical and experimental results. Phys Med Biol.

[19] Bibic A, Knutsson L, Schmidt A, et al (2015) Measurement of vascular water transport in human subjects using time-resolved pulsed arterial spin labelling. NMR Biomed 28:1059–1068. 10.1002/nbm.3344 10.1002/nbm.3344 26147641

[20] Simchon S, Jan KM, Chien S (1987). Influence of reduced red cell deformability on regional blood flow. Am J Physiol.

[21] Driessen GK, Haest CW, Heidtmann H, Kamp D, Schmid-Schonbein H (1980). Effect of reduced red cell “deformability” on flow velocity in capillaries of rat mesentery. Pflugers Arch.

[22] Sosa JM, Nielsen ND, Vignes SM, Chen TG, Shevkoplyas SS (2014). The relationship between red blood cell deformability metrics and perfusion of an artificial microvascular network. Clin Hemorheol Microcirc.

[23] Barshtein G, Arbell D, Yedgar S (2018). Hemodynamic functionality of transfused red blood cells in the microcirculation of blood recipients. Front Physiol.

[24] Rosner B (2016). Fundamentals of biostatistics.

[25] Baskerville TA, McCabe C, Weir CJ, Macrae IM, Holmes WM (2012). Noninvasive MRI measurement of CBF: evaluating an arterial spin labelling sequence with 99mTc-HMPAO CBF autoradiography in a rat stroke model. J Cereb Blood Flow Metab.

[26] Baughman VL, Hoffman WE, Miletich DJ, Albrecht RF (1990). Cerebrovascular and cerebral metabolic effects of N2O in unrestrained rats. Anesthesiology.

[27] Lu H, Scholl CA, Zuo Y, Demny S, Rea W, Stein EA, Yang Y (2010). Registering and analyzing rat fMRI data in the stereotaxic framework by exploiting intrinsic anatomical features. Magn Reson Imaging.

[28] Wells JA, Siow B, Lythgoe MF, Thomas DL (2013). Measuring biexponential transverse relaxation of the ASL signal at 9.4 T to estimate arterial oxygen saturation and the time of exchange of labeled blood water into cortical brain tissue. J Cereb Blood Flow Metab.

[29] Bibic A, Knutsson L, Ståhlberg F, Wirestam R (2010). Denoising of arterial spin labeling data: wavelet-domain filtering compared with Gaussian smoothing. Magn Reson Mater Phys.

[30] Tsekos NV, Zhang FY, Merkle H, Nagayama M, Iadecola C, Kim SG (1998). Quantitative measurements of cerebral blood flow in rats using the FAIR technique: Correlation with previous iodoantipyrine autoradiographic studies. Magn Reson Med.

[31] James F, Roos M (1975). Minuit - system for function minimization and analysis of parameter errors and correlations. Comput Phys Commun.

[32] Rothman KJ (1990). No adjustments are needed for multiple comparisons. Epidemiology.

[33] Shevkoplyas SS, Yoshida T, Gifford SC, Bitensky MW (2006). Direct measurement of the impact of impaired erythrocyte deformability on microvascular network perfusion in a microfluidic device. Lab Chip.

[34] Strandgaard S, MacKenzie ET, Jones JV, Harper AM (1976). Studies on the cerebral circulation of the baboon in acutely induced hypertension. Stroke.

[35] Cole DJ, Drummond JC, Patel PM, Marcantonio S (1994). Effects of viscosity and oxygen content on cerebral blood flow in ischemic and normal rat brain. J Neurol Sci.

[36] Hurn PD, Traystman RJ, Shoukas AA, Jones MD (1993). Pial microvascular hemodynamics in anemia. Am J Physiol.

[37] Muizelaar JP, Wei EP, Kontos HA, Becker DP (1986). Cerebral blood flow is regulated by changes in blood pressure and in blood viscosity alike. Stroke.

[38] Chen RY, Carlin RD, Simchon S, Jan KM, Chien S (1989). Effects of dextran-induced hyperviscosity on regional blood flow and hemodynamics in dogs. Am J Physiol.

[39] Fan FC, Chen RY, Schuessler GB, Chien S (1980). Effects of hematocrit variations on regional hemodynamics and oxygen transport in the dog. Am J Physiol.

[40] Aarts PA, Heethaar RM, Sixma JJ (1984). Red blood cell deformability influences platelets–vessel wall interaction in flowing blood. Blood.

[41] Thomas DL, Lythgoe MF, van der Weerd L, Ordidge RJ, Gadian DG (2006). Regional variation of cerebral blood flow and arterial transit time in the normal and hypoperfused rat brain measured using continuous arterial spin labeling MRI. J Cereb Blood Flow Metab.

[42] Silva AC, Kim SG, Garwood M (2000). Imaging blood flow in brain tumors using arterial spin labeling. Magn Reson Med.

[43] Ji Y, Lu D, Jiang Y, Wang X, Meng Y, Sun PZ (2021). Development of fast multi-slice apparent T_1_ mapping for improved arterial spin labeling MRI measurement of cerebral blood flow. Magn Reson Med.

[44] Wang X, Zhu XH, Zhang Y, Chen W (2015) Simultaneous imaging of CBF change and BOLD with saturation-recovery-T1 method. PLoS One 10:e0122563. 10.1371/journal.pone.0122563 10.1371/journal.pone.0122563 PMC4408048 25905715

[45] Kety SS, Schmidt CF (1948). The effects of altered arterial tensions of carbon dioxide and oxygen on cerebral blood flow and cerebral oxygen consumption of normal young men. J Clin Invest.

[46] Pulsinelli WA, Brierley JB (1979). A new model of bilateral hemispheric ischemia in the unanesthetized rat. Stroke.

[47] Rao SM, Salmeron BJ, Durgerian S, Janowiak JA, Fischer M, Risinger RC, Conant LL, Stein EA (2000). Effects of methylphenidate on functional MRI blood-oxygen-level-dependent contrast. Am J Psychiatry.

[48] Johnson CS, Verdegem TD (1988). Pulmonary complications of sickle-cell disease. Semin Respir Med.

[49] Powars DR (1990). Sickle cell anemia and major organ failure. Hemoglobin.

[50] Chen Y, Wang DJ, Detre JA (2011). Test-retest reliability of arterial spin labeling with common labeling strategies. J Magn Reson Imaging.

[51] Sicard K, Shen Q, Brevard ME, Sullivan R, Ferris CF, King JA, Duong TQ (2003). Regional cerebral blood flow and BOLD responses in conscious and anesthetized rats under basal and hypercapnic conditions: implications for functional MRI studies. J Cereb Blood Flow Metab.

[52] Steward CA, Marsden CA, Prior MJ, Morris PG, Shah YB (2005). Methodological considerations in rat brain BOLD contrast pharmacological MRI. Psychopharmacology (Berl).

[53] An X, Mohandas N (2008). Disorders of red cell membrane. Br J Haematol.

[54] Safeukui I, Buffet PA, Deplaine G, Perrot S, Brousse V, Ndour A, Nguyen M, Mercereau-Puijalon O, David PH, Milon G, Mohandas N (2012). Quantitative assessment of sensing and sequestration of spherocytic erythrocytes by the human spleen. Blood.

[55] Dondorp AM, Kager PA, Vreeken J, White NJ (2000). Abnormal blood flow and red blood cell deformability in severe malaria. Parasitol Today.

[56] Moutzouri AG, Athanassiou GA, Dimitropoulou D, Skoutelis AT, Gogos CA (2008). Severe sepsis and diabetes mellitus have additive effects on red blood cell deformability. J Infect.

[57] Tomaiuolo G (2014). Biomechanical properties of red blood cells in health and disease towards microfluidics. Biomicrofluidics.

[58] Caprari P, Massimi S, Diana L, Sorrentino F, Maffei L, Materazzi S, Risoluti R (2019). Hemorheological alterations and oxidative damage in sickle cell anemia. Front Mol Biosci.

