# Supplementary material for: Effects of red blood cells with reduced deformability on cerebral blood flow and vascular water transport: measurements in rats using time-resolved pulsed arterial spin labelling at 9.4 T
Source: Eur Radiol Exp. 2021 Dec 21;5:53. doi: 10.1186/s41747-021-00243-z (PMC8692551; doi:10.1186/s41747-021-00243-z)
Supplement: Supplementary file 1 — Additional file 1: Table S1. Absolute mean CBF and CTT values, and the corresponding standard deviations, for all investigated regions of interest and all points in time, for control group (CG) and study group (SG). [file 41747_2021_243_MOESM1_ESM.docx]

## **ELECTRONIC SUPPLEMENTARY MATERIAL**

## **Appendix 1**

Consider the Fokker-Planck equation (A1)

$\frac{\partial c}{\partial t}=-F\frac{\partial c}{\partial V}+P\frac{\partial^{2}c}{\partial V^{2}}-\frac{c}{T_{1}}, V\in R, t\geq0$ (A1)

and its bolus-tracking solution (A2) (i.e., Eq. 19 in Ref. [18]):

$c\left( V,t \right)=\int_{0}^{t} c_{0}(\tau)\frac{\exp\left( -\frac{t-\tau}{T_{1}} \right)}{\sqrt{4\pi P \left( t-\tau\right)}}\frac{V}{\left( t-\tau\right)}exp\left( -\frac{{(V-F\left( t-\tau\right))}^{2}}{4 P(t-\tau)} \right)d\tau$ (A2)

where F is blood flow and P represents the diffusion component (i.e., pseudo-diffusion and the filtration through the blood-brain barrier). With the following boundary conditions,

$c\left( 0,t \right)=c_{a}\left( t \right)=0$ for $t<0$ and $t>t_{b}$,

the solution to (A2) becomes

$c\left( V,t \right)=\frac{V}{\sqrt{4\pi P}}\int_{0}^{min(t,t_{b})} c_{a}\left( \tau\right)\frac{\exp\left( -\frac{t-\tau}{T_{1}} \right)}{\left( t-\tau\right)^{\frac{3}{2}}}exp\left( -\frac{{(V-F\left( t-\tau\right))}^{2}}{4 P(t-\tau)} \right)d\tau$ (A3)

By defining the relative total transit time rTTT $=\frac{V}{F}$ , $CTT=\frac{P}{F^{2}}$ , $\frac{F^{2}}{P}=\frac{1}{CTT}$ and $\frac{V^{2}}{P}=\frac{F^{2}\cdot{rTTT}^{2}}{F^{2}\cdot CTT}=\frac{{rTTT}^{2}}{CTT}$, then the general solution will be given as

$c\left( t,rTTT,CTT \right)=\frac{rTTT\cdot S}{\sqrt{4 \pi CTT}}\int_{0}^{min(t,t_{b})} c_{a}\left( \tau\right)\frac{\exp\left( -\frac{t-\tau}{T_{1}} \right)}{\left( t-\tau\right)^{\frac{3}{2}}}exp\left( -\frac{\left( rTTT-\left( t-\tau\right) \right)^{2}}{4 CTT\left( t-\tau\right)} \right)d\tau$ (A4)

where *S* is a scaling factor aiming to correct for proportional errors in the registration of arterial concentration, such as partial volume effects, M_0_ differences and different T_2_ in blood and tissue.
